# Distinct degree of radiculopathy at different levels of peripheral nerve injury

**DOI:** 10.1186/1744-8069-8-31

**Published:** 2012-04-26

**Authors:** Noboru Takiguchi, Munehito Yoshida, Wataru Taniguchi, Hiroshi Hashizume, Hiroshi Yamada, Nobuyuki Miyazaki, Naoko Nishio, Terumasa Nakatsuka

**Affiliations:** 1 Department of Orthopaedic Surgery, Wakayama Medical University, 811-1 Kimiidera, Wakayama, 641-8509, Japan; 2 Pain Research Center, Kansai University of Health Sciences, 2-11-1 Wakaba Kumatori Sennan, Osaka, 590-0482, Japan

**Keywords:** Radiculopathy, Microglia, Patch-clamp

## Abstract

**Background:**

Lumbar radiculopathy is a common clinical problem, characterized by dorsal root ganglion (DRG) injury and neural hyperactivity causing intense pain. However, the mechanisms involved in DRG injury have not been fully elucidated. Furthermore, little is known about the degree of radiculopathy at the various levels of nerve injury. The purpose of this study is to compare the degree of radiculopathy injury at the DRG and radiculopathy injury proximal or distal to the DRG.

**Results:**

The lumbar radiculopathy rat model was created by ligating the L5 nerve root 2 mm proximal to the DRG or 2 mm distal to the DRG with 6.0 silk. We examined the degree of the radiculopathy using different points of mechanical sensitivity, immunohistochemistry and *in vivo* patch-clamp recordings, 7 days after surgery. The rats injured distal to the DRG were more sensitive than those rats injured proximal to the DRG in the behavioral study. The number of activated microglia in laminas I–II of the L5 segmental level was significantly increased in rats injured distal to the DRG when compared with rats injured proximal to the DRG. The amplitudes and frequencies of EPSC in the rats injured distal to the DRG were higher than those injured proximal to the DRG. The results indicated that there is a different degree of radiculopathy at the distal level of nerve injury.

**Conclusions:**

Our study examined the degree of radiculopathy at different levels of nerve injury. Severe radiculopathy occurred in rats injured distal to the DRG when compared with rats injured proximal to the DRG. This finding helps to correctly diagnose a radiculopathy.

## Background

There are many patients who suffer from radiculopathy, characterized by spontaneous pain, weakness and numbness in the buttock, leg, and foot and difficulty in controlling specific muscles. Radiculopathy can occur in any part of the spine, most commonly in the lower back (lumbar radiculopathy) and neck (cervical radiculopathy) and not in the middle of the spine (thoracic radiculopathy). Radiculopathy is caused by compression or irritation of the spinal nerves. This can be due to mechanical compression of the nerve by a disc herniation or thickening of surrounding ligaments. Other causes of radiculopathy include diabetes, which can decrease the normal blood flow to the spinal nerves. Inflammation from trauma can also lead to radiculopathy from direct irritation of the nerves.

Radiculopathy is thought to be caused by a series of changes in the sensory processing system, functional reorganization of sensory transmission and development of neural plasticity, in both the peripheral and central nervous systems. Basic research has tended to focus on nerve injury preventing spinal cord neurons from receiving sensory information and relaying it to the brain. The superficial dorsal horn, especially the substantia gelatinosa (SG; lamina horn), plays an important role in modulating nociceptive transmission [1]. In previous studies, whole cell patch-clamp techniques have been adapted to SG neurons in a spinal cord slice with an attached dorsal root to investigate synaptic responses to peripheral nerve stimulation [2,3]. These studies revealed that SG neurons exhibit a variety of excitatory synaptic responses, however it remains to be settled what kinds of stimulation applied to the skin elicit these responses.

Wind-up is a progressive, frequency-dependent facilitation of neuronal responses induced by repetitive electrical stimulation of afferent C-fibers [4]. Long-term potentiation (LTP) of excitatory synaptic transmission is a long-lasting enhancement in signal transmission between two neurons that results from stimulating them synchronously. It is one of several phenomena underlying synaptic plasticity, the ability of chemical synapses to change their strength. LTP is synaptic plasticity not only in the peripheral nervous system and brain, but also in the spinal cord [5]. The cellular mechanisms of central sensitization and its relationship to the hypersensitivity and hyperalgesia of radiculopathy are still not fully elucidated. Previous research has used *in vivo* patch-clamp techniques to analyze excitatory synaptic responses evoked by cutaneous mechanical stimuli [6,7]. An *in vivo* preparation of a rat spinal cord was used to investigate the superficial dorsal horn neuron response to naturally applied noxious cutaneous stimuli, offering a more comprehensive study of nociceptive processing in the rats superficial dorsal horn.

Nerve injury produces the activation of not only neurons but also glial cells in the central nervous system (CNS) [8-10]. Glial cells make up over 70% of the total cell population in the CNS and are classified into astrocytes, oligodendrocytes, and microglia. Microglia activation following nerve injury is significantly increased compared with oligodendrocytes and astrocytes. Microgliosis (accumulation of activated microglia) around degenerative neurons is a common pathological feature of various neurological disorders including radiculopathy. Microglia exhibit a common, long-term response to a wide range of stimuli that threaten physiological homeostasis. This response includes changes in morphology, gene expression, function and number. Peripheral nerve injury leads to dramatic activation of microglia within the spinal dorsal horn [11]. Microglia activation in the spinal cord progresses through a hypertrophic morphology, with thickened and retracted processes and an increase in cell number. These criteria are immunohistochemical markers for assessing the activation state of microglia *in vivo* and among them, the change in cell number is the most prominent event [12]. Peripheral nerve injury increases the number of dorsal horn microglia by two to four fold [13-17].

Lumbar disc herniation occurs mostly in the spinal canal, because of injury proximal to the dorsal root ganglion (DRG). However, sometimes we face a specific type of radiculopathy, in which the percentage of lumbar disc herniation in the far lateral zone was 4.4–11.7% [18-20]. But the spinal nerve mechanisms around the DRG seem to be obscure, with little known about the degrees of radiculopathy injured at the DRG and proximal or distal to the DRG. Radiculopathy is an important and largely unresolved medical problem that requires further research into the etiological factors, to determine the correct diagnosis.

The purpose of this study was to examine the degrees of radiculopathy following injury at the DRG and proximal or distal to the DRG using different points of mechanical sensitivity, immunohistochemistry and *in vivo* patch-clamp recordings.

## Results

### Mechanical sensitivity

We counted the numbers of withdrawal reflexes in response to a sequential series of 10 tactile stimulations to the plantar surface of the ipsilateral (nerve root injured) hind paw using a 10 g von Frey filament. As rats rarely responded to the mechanical stimuli prior to surgery, the elevated behavioral responses evident after surgery were defined as allodynia. Behavioral tests were performed 7 days (7.8 ± 2.1 days, 220.5 ± 21.6 g) after surgery.

**Figure 1:**
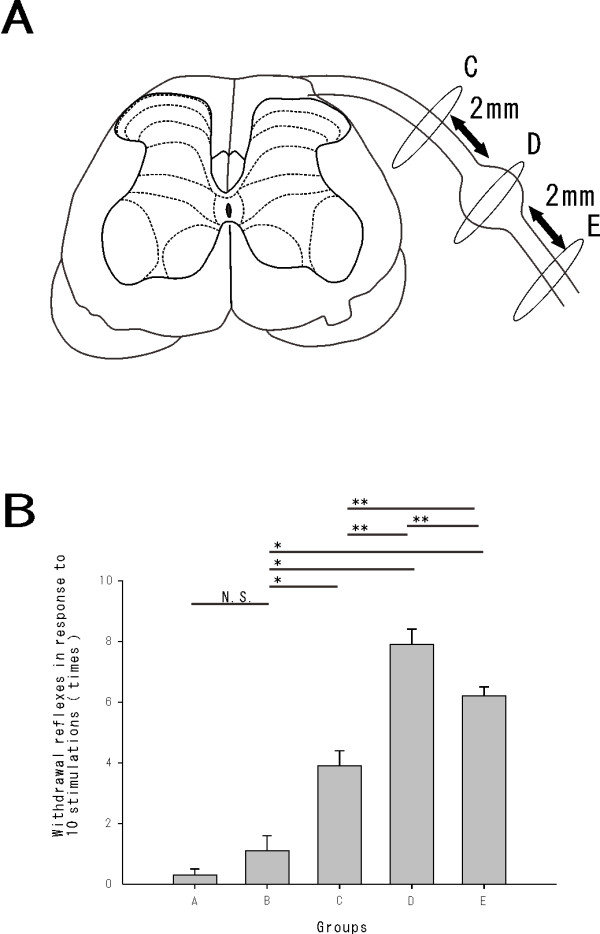
***A.*Schematic diagram of the radiculopathy rat model from the L5 nerve to the spinal cord: The L5 nerve was ligated at 2 mm proximal to the DRG (group C), at the DRG (group D), at 2 mm distal to the DRG (group E) by a microscope at 5× magnification.***B.* Withdrawal reflexes to 10 stimulations of a 10 g von Frey filament: The withdrawal threshold was a significantly different for nerve injury distal to the DRG compared with injury proximal to the DRG. There were no significant differences in the number of withdrawal reflexes between normal group A and the sham group B. The nerve injury groups (C, D, E) were more sensitive than the sham group (*P < 0.001), with significant differences between groups C, D and E (**P < 0.001).

The number of withdrawal reflexes was 0.3 ± 0.2 times for group A (n = 10), 1.1 ± 0.5 times for group B (n = 10), 3.9 ± 0.5 times for group C (n = 10), 7.9 ± 0.5 times for group D (n = 10) and 6.2 ± 0.3 times for group E (n = 10). The number of withdrawal reflexes in the sham group (B) was not significantly increased compared with that in the normal group (A). While the rats in the nerve injury groups (C, D, and E) were significantly more sensitive compared with the rats in the sham groups (Student’s *t* test, *P* < 0.001). The rats in group D were the most hypersensitive in the operated group, followed by groups E and C (*P* < 0.001) [Figure 1B].

### Immunohistochemistry

**Figure 2:**
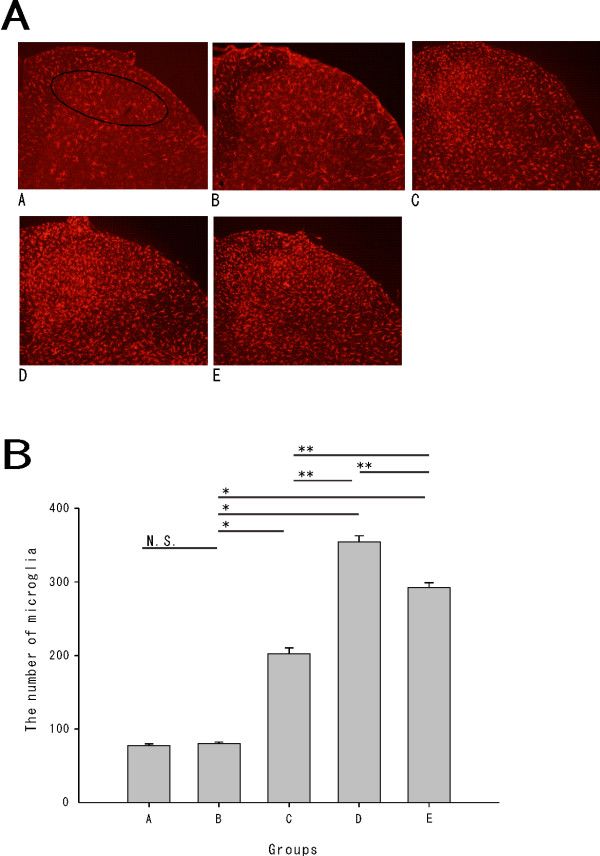
***A.*Iba1 positive microglia in laminas I–II of the injured L5 segmental level: Activated microglia in the nerve injury groups (C, D, E) were observed, compared with the normal (A) and sham group (B).***B.* The total number of microglia in laminas I–II of the injured L5 segmental level : There was no significant difference in the number of activated microglia between groups A and B. The nerve injury groups (C, D, E) were more sensitive than the sham group (*P < 0.001), with significant differences between C, D and E (**P < 0.001).

We used a polyclonal antiserum directed against the ionized calcium-binding adapter molecule 1 (Iba1) to investigate whether microglia was activated and increased in the spinal dorsal horn 7 days after the surgery (208.4 ± 21.7 g) [Figure 2A]. We counted the number of microglia in laminas I–II of the L5 segment.

The number of activated microglia evident was 77.2 ± 2.5 for group A (n = 5), 80.0 ± 2.1 for group B (n = 5), 202.4 ± 8.0 for group C (n = 5), 354.6 ± 8.1 for group D (n = 5) and 292.1 ± 6.6 for group E (n = 5). There was no significant difference between groups A and B. The number of activated microglia was significantly increased in the nerve injury groups compared with the sham group (*P* < 0.001). The activated microglia seen in the nerve injury groups (C, D, E) developed at the superficial dorsal horn. The number of activated microglia was most significant in group D, followed by groups E and C (*P* < 0.001) [Figure 2B].

### *In vivo* patch-clamp recordings

**Figure 3:**
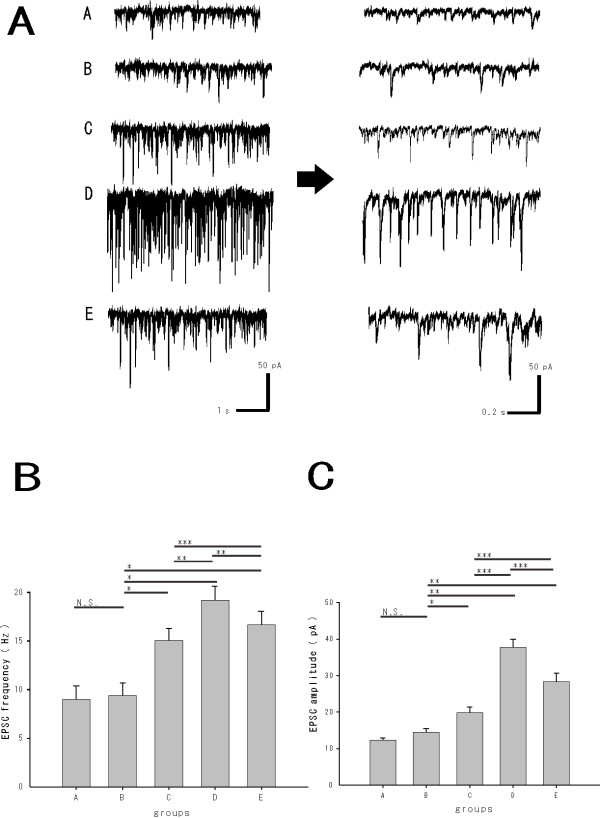
***A. In vivo*patch clamp data: EPSC were recorded at the SG of the injured L5 segmental level.** All SG neurons were recorded at a holding potential (V_H_) of −70 mV, where no IPSC were observed. *B.* The average frequencies of EPSC in laminas I–II of the L5 segmental level: No significant difference in EPSC frequency was shown between groups A and B. The frequencies of EPSC in nerve injury group (C, D, E) were significantly increased compared with the sham group (*P < 0.001) and interestingly there were significant differences between groups C, D and E (**P < 0.001 and ***P < 0.05, respectively). *C.* The average amplitudes of EPSC in laminas I–II of the L5 segmental level: No significant difference in the mean amplitudes of EPSC was observed between groups A and B. There were significant differences in mean amplitudes of EPSC between the nerve injury groups (C, D, E) and the sham group (B) (*P < 0.05 and **P < 0.001, respectively) as well as significant differences between groups C, D and E (**P < 0.001).

Rats (218.4 ± 23.5 g n = 45) developed mechanical hypersensitivity approximately 7 days (7.6 ± 2.4 days) after surgery. A rat spinal cord preparation could be maintained in a stable condition for over 12 hours, which was equivalent to previous patch-clamp experiments using an artificial ventilator. Whole-cell patch-clamp recordings were performed in 50 SG neurons from the L5 segmental level of the spinal cord. All SG neurons were recorded at a holding potential (V_H_) of −70 mV, where no inhibitory postsynaptic currents (IPSC) were observed [Figure 3A].

The average frequencies of excitatory postsynaptic currents (EPSC) were recorded in laminas I–II of the L5 segmental area. The frequencies of EPSC were 9.0 ± 1.4 Hz for group A (n = 10), 9.4 ± 1.3 Hz for Group B (n = 10), 14.6 ± 1.7 Hz for Group C (n = 10), 19.2 ± 2.0 Hz for Group D (n = 10), 17.3 ± 2.1 Hz for Group E (n = 10). The frequencies of EPSC in the nerve injury groups (C, D, E) were significantly increased compared with the sham group (*P* < 0.001). Interestingly, there were significant differences in the frequencies of EPSC between groups C, D and E (*P* < 0.001 and *P* < 0.05, respectively) [Figure 3B].

The amplitudes of EPSC were 12.3 ± 0.6 pA for group A (n = 10), 14.4 ± 1.0 pA for group B (n = 10), 19.8 ± 1.6 pA for group C (n = 10), 37.7 ± 2.2 pA for group D (n = 10) and 28.3 ± 2.4 pA for group E (n = 10). No significant difference in mean amplitudes of EPSC was observed between groups A and B. There were significant differences between groups B and C (*P* < 0.05), B and D (*P* < 0.001) and B and E (*P* < 0.001). Finally, there were significantly differences in mean amplitudes in C, D and E (*P* < 0.001) [Figure 3C].

## Discussion

Several studies have investigated different types and degrees of nerve root or spinal nerve injuries [11,21]. Lesions close to the DRG produce more apoptosis when compared with lesions more distal to the DRG [22]. However, there was no report to compare the degrees of radiculopathy injured proximal or distal to the DRG. We investigated the degrees of radiculopathy following nerve injury via nerve ligation at an equal distance (2 mm proximal or distal) to the DRG. The degrees of radiculopathy were assessed using different points of mechanical sensitivity, immunohistochemistry and *in vivo* patch-clamp recordings.

Mechanical allodynia was observed at the plantar surface of the ipsilateral (nerve root injury site) hind paw at 7 days after surgery. The withdrawal threshold to von Frey stimulation in the nerve injury groups decreased compared with the sham model rats. Our behavioral data were consistent with the previous studies that showed development of maximum allodynia to occur at around 7 days post-injury in a rat model of nerve injury [23,24]. Interestingly, there was a significant difference in withdrawal threshold between the proximal and distal nerve injury to the DRG in this study.

Resting microglia (in the normal state) act as sensors for stimuli that threaten physiological homeostasis, including CNS trauma, ischemia, infection and neurodegeneration. Once activated by these stimuli, microglia undergo a common, series of progressive changes in morphology, function and number [13,25], which have been implicated in inducing central sensitization of spinal neurons [26]. ATP [27], substance P [28] and glutamate [29] are released in high amounts during central sensitization and participate in its induction and activate microglia [13,25], so that the sensitization of dorsal horn neurons may also stimulate microglia, which become further activated, establishing a feed-forward cycle. Therefore, microglia is closely associated with the mechanisms of radiculopathy, with the number of activated microglia acting as an index for radiculopathy [13]. In this study, we show an approximate fourfold increase in microglia activation when compared with sham rats after surgery. Results consistent with previous studies, which found nerve injury to cause a two- to four-,fold increase in the number of microglia in the dorsal horn [13-17]. Interestingly, we show the number of microglia in rats injured distal to the DRG was significantly increased when compared with rats injured proximal to the DRG, consistent with the mechanical sensitivity results in the behavioral study. Together, these results indicate a difference in the degree of radiculopathy at distinct levels of nerve injury.

Microglia is also closely associated with neurons and astrocytes. Astrocytes have a more direct and active role in glutamatergic synapse function by releasing glutamate [30], releasing modulatory substances [31] and expressing functional N-Methyl D-Aspartate (NMDA) receptors [32]. NMDA receptors are activated by the wind-up phenomena, which is central pain sensitization caused by repeated stimulation of peripheral nerve fibers, leading to stimulation of C-fibers and a progressively increasing electrical response in the corresponding superficial dorsal horn. The mechanism underlying this phenomenon involves the release of glutamate by these pathologically sensitized C-fibers. The glutamate interacts with the postsynaptic NMDA receptors, which aids the sensitization of the dorsal horn. Presynaptic neuronal voltage-gated sodium calcium channels are largely responsible for the release of this glutamate as well as the neuropeptide substance P [33]. Microglia also express α-amino-3-hydroxy-5-methyl-4-isoxazolepropionic acid/kainate (AMPA/KA) receptors that mediate the release of inflammatory cytokines such as tumor necrosis factor-α (TNF-α) [34]. AMPA receptors are glutamate receptors that are integral to plasticity and synaptic transmission at many postsynaptic membranes. One of the investigated forms of plasticity in the nervous system is long-term potentiation LTP. LTP explained that glutamate was bound to postsynaptic AMPA receptors. Ligand binding causes AMPA receptors to open and for sodium ions to flow into the postsynaptic cells, resulting in a depolarization. Therefore, we assumed that microglia participated in the changes of glutamatergic synaptic transmission observed at the nerve injury. Receptors expressed on microglial membranes are thought to allow sensing of neuronal activity and/or communication with astrocytes [35]. Glutamate neurotransmission has been associated with pain processing at multiple levels of the neuroaxis [36]. The connections between the C-fibers and the SG neurons play a critical role in pain sensation through an action of the neuropeptides. In the spinal dorsal horn neurons, activation of fine-afferent fibers produce a variety of synaptic events which are likely to be mediated by a number of neurotransmitters, including excitatory amino acids and neuropeptides. We examined the synaptic activities in the superficial dorsal horn neurons response to nerve injury using *in vivo* patch clamp methods. We show EPSC amplitudes were significantly larger following injury distal to the DRG compared with injury proximal to the DRG. In a previous study, EPSC amplitudes obtained from SG neurons at the L5 segmental level of the spinal cord after nerve injury was larger than the sham-operated animals [37]. Interestingly, this is the first study to show significant differences in excitatory synaptic activities in a nerve injury proximal and distal to the DRG. This result also indicates a marked difference in the degree of radiculopathy at distinct points of nerve injuries.

In general, the direction of information flow from the periphery to the DRG to the spinal cord itself is a main factor in the distal lesion giving rise to stronger neuropathic signs. Thus the changes from peripheral inputs are more critical for neuropathy. It has been demonstrated that radiculopathy is caused by changes in the expression and function of receptors and voltage-dependent sodium channels in peripheral nerves and DRG neurons, as well as at synapses in the nociceptive pathway in the CNS [38,39]. In particular, tetrodotoxin resistant (TTX-r) sodium channels are closely related to radiculopathy. The NaV 1.8, which is one of TTX-r sodium channel, is important in the neurophysiological and behavioral effects [40,41]. The NaV 1.8 sodium channels are expressed in both A- and C-fiber populations [42,43] and differentially regulated after peripheral nerve injury, and selectively distributed in peripheral sensory neurons [44]. Sodium channels are involved in the propagation of action potentials, while glutamate receptors expressed on presynaptic terminals of primary afferents in the dorsal horn regulate the release of neurotransmitters. After peripheral nerve injury, injured and uninjured DRG neurons become excitable and exhibit ectopic firing [45,46]. When the spinal nerve is injured distal to the DRG, the DRG neurons become excitable and exhibit ectopic firing, resulting in intense radiculopathy. In this study, there were differences between the degrees of radiculopathy injured proximal or distal to the DRG. This difference of the nerve injury level might have affected the activation and number of sodium channels to which the dorsal root projects. This may have led to activation of glutamatergic transmission, resulting in activation of AMPA receptors and NMDA receptors. As described above, there were differences in the degrees of radiculopathy.

One possible reason for the difference in the degree of radiculopathy at distinct nerve injury points may be due to changes in blood flow. The blood flow in the nerve root is affected by root constriction [47]. The blood flow supply in the nerve root proximal to the DRG is greater compared with blood flow distal to the DRG [48]. Nerve roots are surrounded by cerebrospinal fluid but not the spinal or peripheral nerves. Spinal nerve roots receive 58% of their nutritional supply from cerebrospinal fluid and 38% from intramural blood vessels, whereas peripheral nerves receive 95% of their nutritional supply from intramural blood vessels [49]. Another possible reason for the difference in the degree of radiculopathy may be the number of apoptotic neurons in the spinal cord. Expression of apoptosis in the spinal cord was found to be associated with radiculopathy after spinal nerve or nerve root injuries [50]. The percentage of apoptosis in the DRG was significantly increased in the distal crush group compared with the proximal crush group. This difference in response was because the DRG was not injured directly, resulting in apoptosis secondary to the nerve injuries [51].

## Conclusion

We investigated the degree of radiculopathy using different points of mechanical sensitivity, immunohistochemistry and *in vivo* patch-clamp recordings. Our study indicated that there was a more intense radiculopathy following injury distal to the DRG rather than proximal to the DRG. Indicating the degree of radiculopathy is dependent on the level of nerve injury.

## Materials and methods

A total of 125 adult male Sprague–Dawley rats weighing 180–210 g were used in this study. Animals were housed in plastic cages at room temperature on a 12-h light/dark cycle with free access to food and water. All animal experimental procedures were approved by the Ethics Committee on Animal Experiments, Wakayama Medical University and were performed in accordance with the UK Animals (Scientific Procedures) Act of 1986 and associated guidelines.

### Surgical protocol for the radiculopathy model

The surgical protocol was modified from the lumbar radiculopathy model, as previously reported [52-54]. Rats were divided into five groups at random: Normal (group A), sham (group B), ligated proximal to the DRG (group C), ligated at the DRG (group D), ligated distal to the DRG (group E). The rats were anesthetized with sodium pentobarbital (50 mg/kg, i.p.), placed into a prone position, and received an incision to the middle of the spine at the L4-L6 level. The paraspinal muscles were retracted to expose the right laminas and facet joints between the L4 and L6 vertebrae. A right L5 hemilaminectomy and foraminotomy was performed. The right L5 nerve and DRG were carefully exposed not to influence the electrical properties. The injury was created by ligating the nerve root 2 mm proximal to the DRG (group C), at the DRG (group D) and at the spinal nerve 2 mm distal to the DRG (group E) using 6.0 silk sutures. [Figure 1A] Great care was taken to ligate such that the diameter of the nerve was seen to be just ligated by a microscope at 5× magnification. The desired degree of constriction retarded, but did not arrest, circulation through the superficial epineural vasculature and sometimes produced a small, brief twitch in the muscle surrounding the exposure [54]. After enough washing, the incision was closed in layers. Rats were allowed to recover in their normal environment. Two groups of control rats were used; One was not operated on (group A), the other received sham procedures, a right L5 nerve root exposure without ligation (group B).

### Mechanical sensitivity

The hind paw withdrawal threshold was measured as the frequency of foot withdrawals elicited by a defined mechanical stimulus using a 10 g von Frey filament [55,56]. The rats were placed in a chamber, measuring 18 × 25 × 18 cm above a wire mesh floor. They were acclimatized to the environment and investigator for at least 20 minutes before the test. A evaluation was performed approximately 7 days (7.8 ± 2.1 days, 220.5 ± 21.6 g, n = 50) after surgery.

The mechanical stimulus was applied to the middle area between the footpads on the plantar surface of the ipsilateral (nerve root injury site) hind paw and maintained for approximately 2 seconds. A withdrawal response was considered valid only if the hind paw was removed completely from the platform. If a rat walked immediately after stimulation of a hair instead of lifting the paw, the hair was reapplied. A trial consisted of application of a von Frey hair to the hind paw five times at 5-second intervals. The hind paw was probed consecutively with 10 stimulations. The trial was repeated 3 times with at least a 10-minute interval. Mechanical sensitivity was evaluated as the frequency of withdrawal responses, expressed as the mean frequency of responses.

Differences between groups were compared using Student’s *t* test or a one-way analysis of variance (ANOVA). Data were presented as mean ± SEM. When ANOVA showed a significant difference, pair-wise comparisons between means were tested by the *post-hoc* Tukey method. Hind paw withdrawal threshold values between groups were considered significantly different with a *P* value < 0.05.

### Immunohistochemistry

Spinal sections were processed for immunohistochemistry using the immunofluorescence [57,58].

At 7 days post-surgery, rats (208.4 ± 21.7 g, n = 25) were perfused through the ascending aorta with saline followed by 4% paraformaldehyde with 1.5% picric acid in 0.16 M phosphate buffer, pH7.2–7.4 (4°C). After perfusion, spinal cords were removed and the L4-L5 spinal cord segments were dissected, post fixed in the same perfusion fixative for 4 hours and then 15% sucrose overnight. All of the spinal cords for each experiment were arranged on the same blocks with optimal cutting temperature embedding medium and mounted on the same slides after sectioning. Transverse spinal sections (15 μm) were cut on a cryostat and processed for immunostaining. Spinal sections were blocked with 2% goat serum in 0.3% Triton X-100 for 1 hour at room temperature and incubated overnight at 4°C with rabbit (polyclonal) antiserum directed against the ionized calcium binding adapter molecule 1 (Iba1; 1:1000; Wako Chemicals, Tokyo, Japan), a marker of microglia [59].

After washing, the sections were then incubated with fluorescent–conjugated secondary antibody (1:1000 Alexa Fluor 594 goat anti-rabbit; Invitrogen, San Diego, CA) for 90 minutes at room temperature.

The immunostained sections were examined and photographed with an Olympus (FSX100, Japan) fluorescence microscope at 40× magnification. Quantification of microglia activation was assessed by the number of Iba1-positive microglia in the lamina I–II of the L5 segment.

Differences between groups were compared using Student’s *t* test or a one-way ANOVA. Data were presented as mean ± SEM. When ANOVA showed a significant difference, pair-wise comparisons between means were tested by the *post-hoc* Tukey method. Values were considered statistically significant with a *P* value < 0.05.

### *In vivo* patch-clamp recordings

The methods used for the *in vivo* patch-clamp recordings were as described previously [6,7,60,61]. Rats (218.4 ± 23.5 g, n = 45) were assessed approximately 7 days (7.6 ± 2.4 days) post- surgery when mechanical hypersensitivity had developed fully. Rats were anesthetized with urethane (1.2 g/kg, i.p.). Artificial ventilation of the pneumothorax was not performed as the rats could be maintained in good condition by supplying oxygen through a nose cone [60]. If a withdrawal reflex appeared, a supplemental dose of urethane was given during surgery and the data collection period. A heating pad was placed beneath the rat to maintain its body temperature at 37–38°C. The rat was placed in a stereotaxic apparatus (Model STS-B&SR-5R-HT, Narishige, Tokyo, Japan) and lumbar spinal cord at the L5 segmental area was exposed by a thoracolumbar laminectomy at the level from Th12 to L2. The dura was cut under a microscope with 40× magnification and a recording electrode was advanced into the SG from the surface of the spinal cord. The pia-arachnoid membrane of the right L5 dorsal root entry zone was removed allowing the patch electrode to enter the spinal cord. The surface of the spinal cord was irrigated with Krebs solution (10–15 ml/min) and equilibrated with a 95% O_2_, 5% CO_2_ gas mixture (117 mM NaCl, 3.6 mM KCl, 2.5 mM CaCl_2_, 1.2 mM MgCl_2_, 1.2 mM NaH_2_PO_4_, 11 mM glucose and 25 mM NaHCO_3_) through glass pipettes at 36.5 ± 0.5°C. At the end of the experiments, the rats were given an overdose of urethane and killed by exsanguinations.

The patch electrodes were pulled from thin-walled borosilicate glass capillaries (outer diameter of 1.5 mm, TW150F-4, World Precision Instruments, Sarasota, FL, USA) using a p-97 puller (Sutter Instrument, Novato, CA, USA) and filled with a patch-pipette solution that contained: 135 mM K-gluconate, 5 mM KCl, 0.5 mM CaCl_2_, 2 mM MgCl_2_, 5 mM EGTA, 5 mM ATP-Mg and 5 mM Hepes-KOH; pH7.2 for excitatory postsynaptic current (EPSC) recordings. The recording electrode with a resistance of 8-12MΩwas advanced at an angle of 30 degrees into the SG through the pia-arachnoid membrane by a micromanipulator (Model MWS-32 S, Narishige, Tokyo, Japan). A gigaohm seal (resistance of 10GΩ) was formed with neurons at a depth of 30–150 μm from the surface of the spinal cord, the membrane patch ruptured because of a period of negative pressure and the whole-cell patch-clamp recording was initiated. Holding potential was set to −70 mV in voltage-clamp mode and recordings were collected using an Axopatch 200B amplifier in conjunction with a Digidata 1440A A/D converter (Molecular Devices, Sunnyvale, CA, USA) and stored on a computer using pCLAMP 10 data acquisition program (Molecular Devices, Sunnyvale, CA, USA).

Differences between groups were compared using Student’s *t* test or a one-way ANOVA. Data were presented as mean ± SEM. When ANOVA showed a significant difference, pair-wise comparisons between means were tested by the *post-hoc* Tukey method. Values were considered statistically significant with a *P* value < 0.05.

## Abbreviations

DRG, Dorsal root ganglia; SG, The substantia gelatinosa; LTP, Long term potential; CNS, Central nervous system; EPSC, Excitatory postsynaptic currents; IPSC, Inhibitory postsynaptic currents; NMDA receptor, N-methyl D-aspartate receptor; AMPA receptor, α-amino-3-hydroxy-5-methyl-4-isoxazole propionic acid receptor; Iba 1, Anti-ionized calcium binding adapter molecule 1; TTX-r sodium channel, Tetrodotoxin resistant sodium channel.

## Competing interests

The authors declare that they have no competing interests.

## Authors’ contributions

All authors read and approved the final manuscript. NT performed or contributed to all experiments, analyzed data and drafted the paper. WT and HH contributed to experiments and analysis. NN contributed to experiments. NM and HY participated in the design of the studies. TN and MY conceived and supervised the project and edited the manuscript.
